# The effect of focus size and intensity on stone fragmentation in SWL on a piezoelectric lithotripter

**DOI:** 10.1007/s00345-019-03069-y

**Published:** 2020-01-10

**Authors:** Julian Veser, Victoria Jahrreiss, Christian Seitz, Mehmet Özsoy

**Affiliations:** 1 Department of Urology, Comprehensive Cancer Center, Medical Vienna General Hospital, University of Vienna, Waehringer Guertel 18-20, 1090 Vienna, Austria; 2 Karl Landsteiner SocietyUrology and Andrology, Vienna, Austria

**Keywords:** Focus size, Shock wave therapy, SWL, Urolithiasis, Fragmentation

## Abstract

**Purpose:**

We aim to analyze the efficacy of different focus sizes and the influence of pulse pressure (intensity) during shock wave lithotripsy (SWL) in terms of stone fragmentation.

**Methods:**

Combination of three focal sizes (*F*1 = 2 mm, *F*2 = 4 mm, *F*3 = 8 mm) and 11 output pressure settings (intensity 10–20) of a piezoelectric lithotripter (Wolf PiezoLith 3000) were tested on artificial stones (*n* = 99). The stones were placed within a 2 mm mesh cage. The needed number of shockwaves (SW) to first visible crack, 50% and 100% stone disintegration were recorded.

**Results:**

Similar number of SW’s were observed until the first crack 10, 11 and 11 SW’s for *F*1, *F*2, and *F*3, respectively (*p* > 0,05). The median number of SW needed for 50% stone disintegration was 245 for *F*1 group, 242 for *F*2 group and 656 for *F*3 group. *F*1 vs *F*2 *p* = 0.7, *F*1 vs *F*3 and *F*2 vs *F*3 *p* < 0.05. Similarly, with larger focus size a higher number of shockwaves were necessary for 100% stone disintegration. 894, 877 and 1708 SW’s for *F*1, *F*2 and *F*3, respectively. Only for *F*1 vs *F*3 and *F*2 vs *F*3 (all *p* < 0.05) a statistical difference was observed. These findings were consistent in all different power settings, with an increased difference in lower power levels (≤ 14).

**Conclusions:**

A smaller focus size, as well as a higher peak pressure results in a more effective stone fragmentation. However, these results need to be confirmed in an in vivo setting with multiple parameters interfering the efficacy, like BMI, respiration or stone migration.

## Introduction

Prior to the introduction of shock wave lithotripsy (SWL) in 1982, active stone removal consisted of surgical removal of the urinary stones or mechanical destruction of bladder stones through the urethra. The minimal invasive nature of SWL offered a safe alternative with convincing efficacy, that lead to its wide acceptance among patients and urologists [1, 2]. As the use of SWL spread to other medical fields in the following years, its role in the treatment of urolithiasis was increasingly challenged by ureteroscopy (URS) and percutaneous lithotripsy (PNL). Technical improvements and miniaturization of surgical instruments, introduction and improvement of laser technology and digital imaging, resulted in higher stone-free rates in fewer therapy sessions for URS and PNL when compared to SWL [3]. On the other hand, technical advancements of SWL primarily improved the lithotripter handling and shock wave generation, like replacing the water bath with gel cushions as well as providing a more stable energy output, but they failed to improve the stone-free rate compared to the first-generation lithotripters, as seen in URS or PNL [4, 5].

Even though the underlying physical principle of the shock wave and its disintegrative effect have been investigated in prior publications [6–9], evidence on the interaction of different lithotripter settings as well as different energy sources and their effect on the disintegration capacity of the shock wave is scarce. Recent developments in lithotripter technology allowed further adjustment of therapy settings like multiple focus sizes or shockwave frequency. The latter was subject of many investigations and the optimal frequency of shock waves to maximize the efficacy was defined in a recent meta-analysis by Li et al. [10]. On the other hand, importance of focus size and shockwave intensity is still not fully understood. We therefore performed a study comparing lithotripter settings with varying intensity and focus sizes in an in vitro stone model to analyze their effect on stone disintegration.

## Materials and methods

In this in vitro study, combinations of three focal sizes (*F*1, *F*2, *F*3) and eleven wave output pressure settings (intensity 10–20) of a piezoelectric lithotripter (Wolf PiezoLith 3000 Richard Wolf GmBH, Knittlingen, Germany) were tested. The lateral diameter of the focal zone at − 6 dB, and the maximum shockwave output pressure (Pmax) according to manufacturer information for each focus setting is: *F*1 = 2 mm, 126 MPa; *F*2 = 4 mm, 119 MPa and *F*3 = 8 mm, 48 MPa. The model stones consisted of spheres of activated aluminum with a diameter of 12.7 mm, surface area of 280 m^2^, a bulk density of 720 kg/m^3^ and a chemical stone composition of Al_2_O_3_ 92.5%, SiO_2_ 0.02%, Fe_2_O_3_ 0.3%, Na_2_O 0.3%. (BASF SE, Ludwigshafen am Rhein, Deutschland).

A combination of three focal sizes and eleven wave output pressure settings (intensity 10–20) was used to disintegrate three model stones for each combination. All together 99 model stones were tested, 33 in each focal size setting at a frequency of 90 shockwaves per minute. For every focus size (1−3) and every intensity setting (10–20), three different stones were disintegrated. To avoid bias of potential wear out of the shockwave generator, a sequence of alternating focus sizes with every increase of output pressure was selected.

**Fig. 1:**
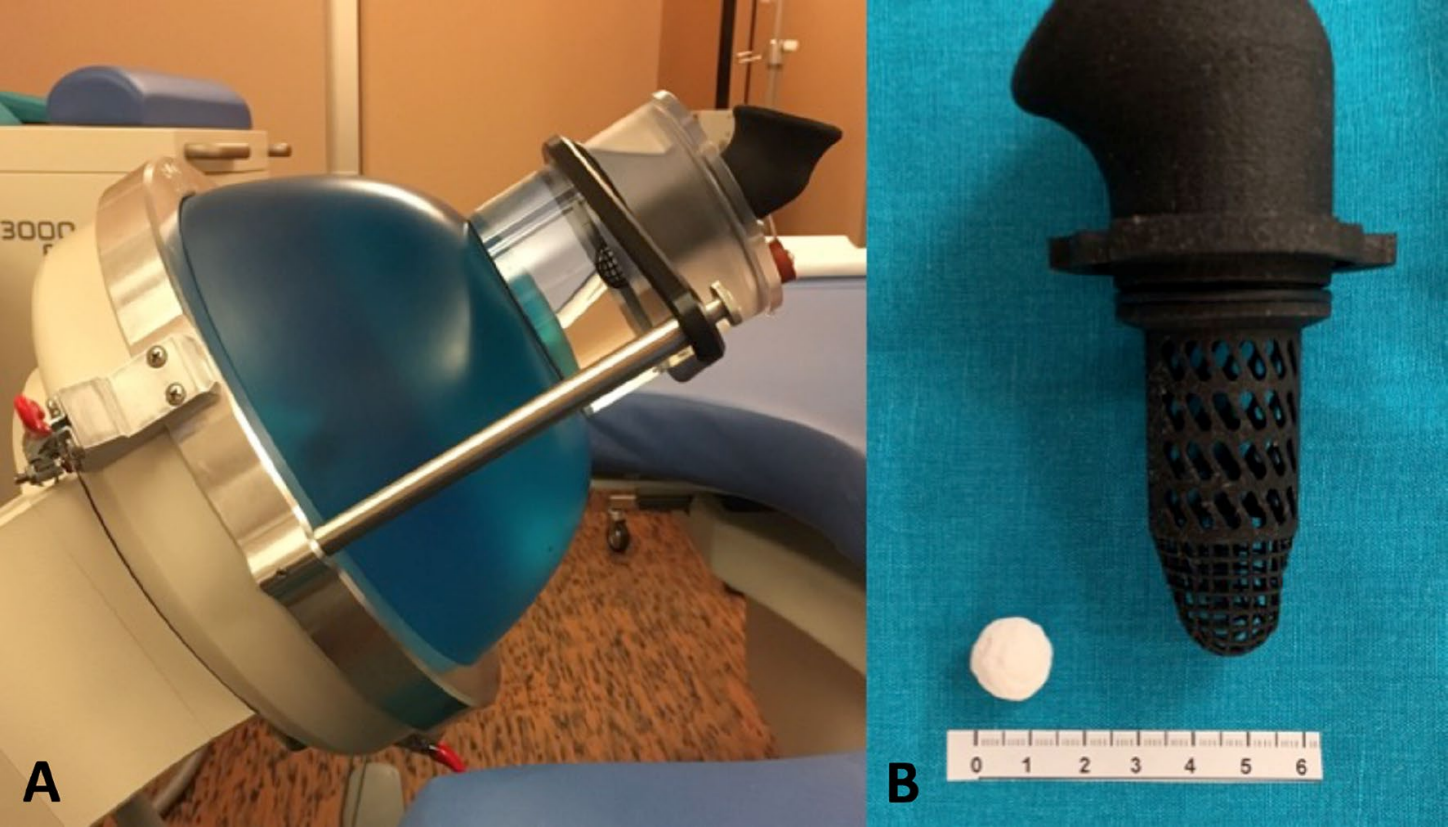
**a** SWL-therapy head with mounted test device. **b** Artificial stone and mesh holder

A test cylinder, containing a 2 mm mesh cage, was installed on to the shockwave source and filled with 500 ml 0.9% NaCl solution. The model stones were placed inside a mesh cage with the mesh acting as a filter through which disintegrated stone fragments passed at a diameter of 2 mm or below, similar to an in vivo model where they would be able to pass freely through the ureter. The mesh cage of the mounted test device held the stone within the geometrical focus of the piezoelectric lithotripter, with a distance of 165 mm to the shock wave generator (Fig. 1).

The needed number of shockwaves to first visible crack, 50% and 100% stone disintegration were recorded. Two independent investigators evaluated 50% disintegration by visual assessment of the stone debris in the mounted cylinder. A predefined line on the cylinder marked the 50% threshold.

### Statistics

Stone disintegration outcomes were checked for standard distribution with Kolmogorov–Smirnov test and non-parametric tests were used for further analysis including Kruskal–Wallis test, Mann–Whitney-*U* test. The relationship between intensity and disintegration was analyzed with Spearman rank correlation coefficient. Stata v.12 (Stata Corp LP, College Station, TX, USA) were used for statistical analysis. All *p* values were two-sided and those < 0.05 were considered statistically significant.

## Results

**Fig. 2:**
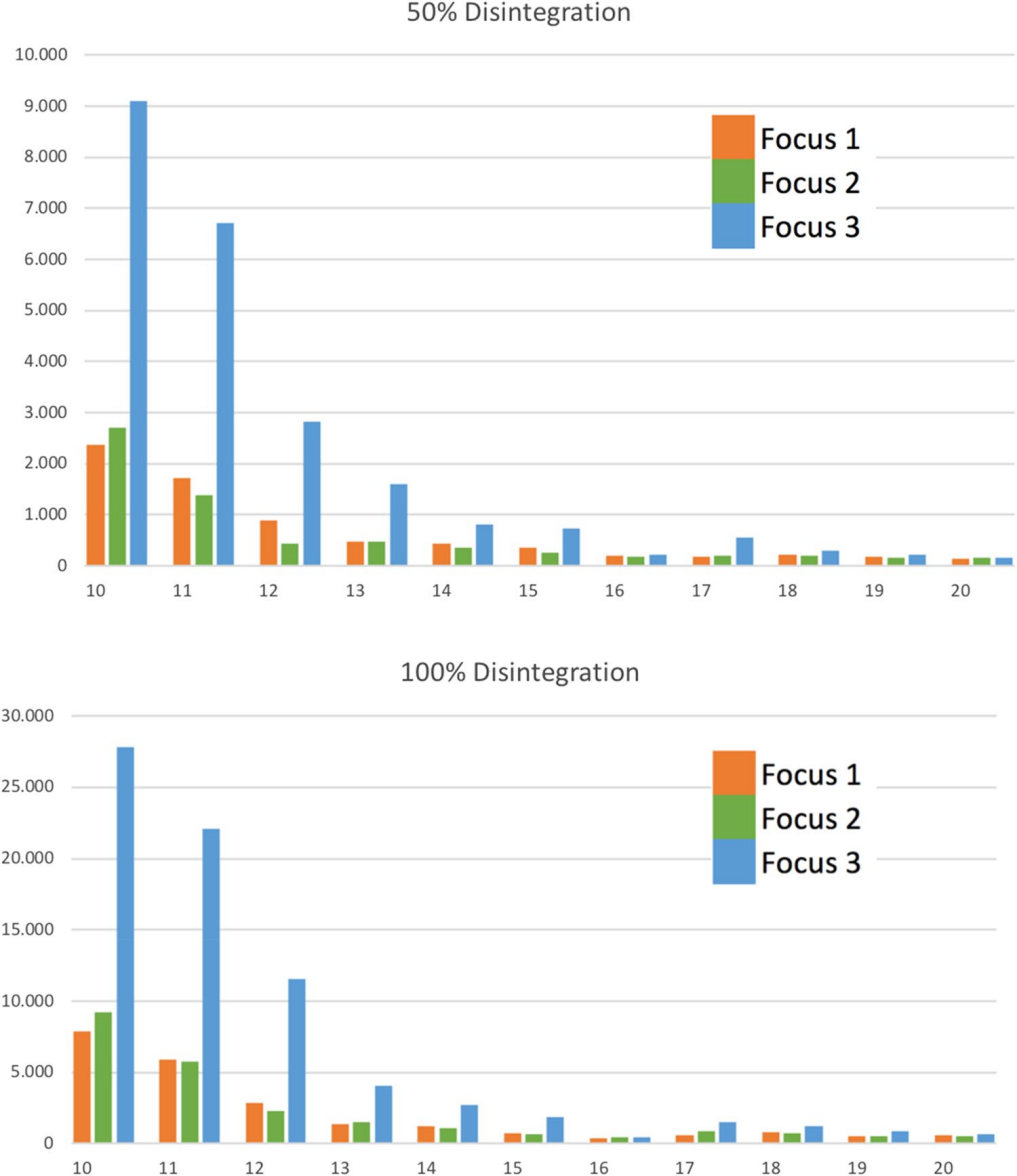
Mean number of shock waves (*y*-axis) for each focus group (*F*1–*F*3, color coded) in correlation to the intensity level (10–20, *x*-axis)

The median number of shockwaves needed until the observation of first crack were 10, 11 and 11 for *F*1, *F*2, and *F*3, respectively, showing no statistical difference (all *p* > 0.05). The median number of shockwaves needed for 50% stone disintegration was 254 for *F*1 group, 242 for *F*2 group and 656 for *F*3 group. There was no statistical difference between the number of necessary shockwaves for 50% stone disintegration between groups *F*1 and *F*2 (*p* = 0.7), whereas a statistical difference was observed for *F*1 vs *F*3 and *F*2 vs *F*3 (all *p* < 0.05). Similarly, with larger focus size a higher number of shockwaves were necessary for 100% stone disintegration. For *F*1 group 894, for *F*2 group 877 and for *F*3 group 1708 median number of shockwaves were needed. Again, no statistical difference was observed between groups *F*1 and *F*2 (*p* = 0.7) on the other hand a statistical difference was observed for *F*1 vs *F*3 and *F*2 vs *F*3 (all *p* < 0.05) (Table 1 and Fig. 2).

**Table 1:** Number of shockwaves needed to achieve 50% and 100% disintegration

	Focus 1 2 mm (*n* = 33)	Focus 2 4 mm (*n* = 33)	Focus 3 8 mm (*n* = 33)
First crack [median (SD)]^a^	10 (± 9.3)	11 (± 42.45)	11 (± 30.17)
50% disintegration			
Shock waves [median (SD)]^b^	254 (877)	242 (871)	656 (3478)
Correlation coefficient with intensity^c^	0.94	0.9	0.92
Focus 1 vs 2 *p* = 0.7, Focus 1 vs and Focus 2 vs 3 *p* < 0.05			
100% disintegration			
Shock waves; [median (SD)]^b^	894 (3085)	877 (3196)	1708 (11,385)
Correlation coefficient with intensity^c^	0.8	0.82	0.86
Focus 1 vs 2 *p* = 0.7, Focus 1 vs and Focus 2 vs 3 *p* < 0.05			

^a^First crack shows the amount of shock waves until the first visible crack of stone

^b^Number of shock waves needed for 50% and 100% disintegration of the stone

^c^Intensity shows a reverse correlation to the number of shock waves needed to disintegrate the stone

A reverse correlation between the shockwave intensity and the number of shockwaves necessary for stone disintegration was shown with a Spearman rank correlation of 0.84 for 50% stone disintegration and 0.73 for 100% stone disintegration (Fig. 2).

## Discussion

Piezoelectric lithotripters use piezoceramic elements mounted on a spherical cylinder which generate a pressure wave through sudden expansion. These acoustic waves are directly focused on an area of interest through their geometrical alignment on the concave carrier, resulting in a high-energy shockwave at the focus point. But as the shockwaves enter the body over a wide area of skin, the patient’s pain level is reduced, allowing SWL without anesthesia. While the average compressive pressure p + (avg) is lower in piezoelectric compared to electrohydraulic or electromagnetic lithotripters, the Wolf Piezolith 3000 uses a double layer of piezoceramic elements to match its power [11]. Through shock wave synchronization of the double-layer piezo-elements, the focal width of the piezoelectric lithotripter can be adjusted to three different focal settings. At the same time, the maximum output pressure (MPa) of the Piezolith 3000 ranges from 48 MPa (Focus 3) to 148 MPa (Focus 1), covering a broader variety when compared to alternative devices. This matches the general development of second and third generation lithotripters, whereas many of the newer devices shifted from a system with a wide focus and low output pressure towards a system with narrow focus and high output pressure [12]. The idea behind was to create an isolated pressure field on the stone with reduced exposure to the surrounding tissue. As the clinical results did not show a significant increase in stone-free rates or decrease in side effects like formation of hematomas, this idea got challenged by others. Eisenmenger reintroduced a wide-focus low-pressure SWL in cooperation with XiXin lithotripters. They designed an electromagnetic lithotripter with a focal size of 18 mm combined with a maximum pressure of 30 MPa. The clinical evaluation showed a total stone-free rate of 86% after 3 months, with a re-treatment rate of 1.24 sessions per patient [13]. These high SFR were explained by an effective fragmentation due to dynamic squeezing of the stone as well as a very slow shockwave frequency of 0.3 Hertz (Hz). Various fragmentation mechanisms have been described previously, but in vitro studies could demonstrate an increased fragmentation rate with binary fragmentation by dynamic squeezing. This is accomplished by a large focus size, leading to wave propagation around the stone boundaries and therefore resulting in circumferential compression. Then again, compression triggers micro-cracks which combine to fractures parallel or vertical to the stress wave due to dynamic fatigue [7, 8, 14].

Qin et al. modified a electrohydraulic HM-3 lithotripter to assess the effect of different focus sizes on stone disintegration. Therefore, they used a reflector insert, which increased the peak positive pressure in the focal plane from 49 to 87 MPa and decreased the − 6 dB focal width from 11 to 4 mm. The acoustic field and pressure distribution produced by the HM-3 using the original reflector in the focal plane (*z* = 0 mm) and a pre-focal plane (*z* = − 15 mm), as well as using the modified reflector in the focal plane, were determined. Three different stone holders were used to mimic different in vivo situations: a mesh holder with a 2 mm grid, a 15 mm finger cot holder and a 30 mm membrane holder mimicking different ways of fragment migration. For all three exposure conditions, similar stone disintegrations rates (∼70%) were observed in the mesh holder after 250 shocks. On the other hand, using the modified reflector significantly lower stone disintegration was observed in the finger cot (45%) or membrane holder (14%) when compared to the corresponding values (56% and 26%) produced by the original reflector. They concluded a superior efficacy in a lithotripter field with low peak pressure and broad focus size. In the mesh holder, similar to our test set-up, fragments ≤ 2 mm could drop out of the focal zone while larger pieces remained in the focal zone, leading to similar results on both focal sizes (3.6 mm vs 10.9 mm). This finding could be explained by attenuation of shock waves caused by accumulating residual fragments as well as an increased dispersion of fragments to a larger area due to high-pressure waves [15].

As stone fragments in vivo either accumulate in the collecting system (especially in the lower and dorsal calyxes) or migrate down the ureter (especially renal pelvis, upper calix and ureteral stones) due to gravity, we used a mesh holder with a 2 mm grid and performed a complete stone disintegration to assess the effect of focus size and intensity on stone fragmentation.

In our setup, focus 1 (2 mm, 126 MPa max) and focus 2 (4 mm, 119 MPa max) have only minor differences, therefore similar disintegration rates were observed. With focus 3 (8 mm, 48 MPa max) on the other hand, a significant higher number of SWs were needed to disintegrate 50% or 100% of the stone. Our presented results therefore oppose the theory of improved fragmentation with wide focus, low pressure SWL. Additionally, our model demonstrates a strong correlation of shock wave power and disintegration rate with a correlation coefficient of 0.84 and 0.73 for 50% and 100% stone disintegration, respectively, meaning a high peak pressure results in a more effective stone destruction.

A comparison between different shock wave generators should be interpreted carefully, this study for example is the first assessment of a piezoelectric device. Even though all lithotripters produce shock waves that have similar wave forms, the amplitude and focal zone of different lithotripters are not the same, and measurements of the properties of the acoustic field can yield very different values. Faragher et al. for example investigated the performance of three different lithotripters, each with a different energy source (electromagnetic, piezoelectric, and electroconductive). They could show that piezoelectric lithotripters produced a near linear increase in mass reduction with the number of shocks delivered. As seen in our study comparing first crack, 50% and 100% disintegration, this represents a gradual erosion by piezoelectric stone treatment. In contrast to the electromagnetic and electroconductive machine, which showed an increase in performance between 500 and 1000 shocks [16]. As the piezoelectric device had a narrow focal zone (2.3 mm) compared to the other two (electroconductive: 6 mm, electromagnetic: 9 mm), the results could be interpreted as different mechanism of stone disintegration, as large focal zones are more likely to produce high internal stress in the stone with an increase of fragmentation over the course of the treatment [8]. But treatment output will always depend on the geometry, size, composition and internal structure of the stone, as well as the characteristics of the lithotripter field. A possible alternative approach to assess the lithotripters efficacy could be a complex calculation of the effective acoustic energy. This represents the energy applied to an area around the stone which should be exceeding a specific threshold for stone disintegration (Eff_12mm_) [17]. Therefore, further basic research is needed to quantify the applied energy by various lithotripters of different designs.

Limitations of our study are the in vitro design with artificial stones, as well as a fixed experimental setup without respiratory movement of the patient. But at the same time this allows very low heterogeneity with results easy to replicate. The bulk density of the used artificial stones represents rather soft human renal calculi, like calcium apatite stones [18]. While limiting the outcomes to a certain stone type, its industrial manufactured standard led to identical stones and therefore comparable results. To assess different artificial stones, representative for other human calculi like calcium-oxalate stones, further studies with different stone compositions are needed.

Furthermore the assessment of 50% stone disintegration was based on a visual evaluation of the stone debris and might be prone to impreciseness. To maximize standardization of the measurement a predefined line on the cylinder to mark the 50% threshold was used, while weighing the stone in between fragmentation was not possible.

Another issue is the width of the large focal zone, with a size only in the average range when compared to different lithotripters available [17]. Especially compared to the XiXin lithotripter, the focal zone is only half the width (8 mm vs 20 mm). The reason for this difference lies in the underlying principle of piezoelectric lithotripters, as the piezoelectric energy source adapts its focus zone by synchronizing two separate layers of piezoceramic elements. Compared to an optical lens in most of the other systems, a wider focal zone leads to far less output pressure and therefore less effective stone fragmentation.

At the same time, this setup has no variation in coupling or stone localization and does not need further manipulation of the lithotripter, which is used in daily clinical practice. Therefore, further studies comparing different focal sizes in vivo are necessary, ultimately helping in the clinical decision-making process when to use which lithotripter setting.

## Conclusion

For piezoelectric lithotripsy, a smaller focus size of 2–4 mm lateral diameter at − 6 dB as well as a higher peak pressure results in a more effective in vitro stone fragmentation. However, these results need to be confirmed in an in vivo setting with multiple parameters interfering the efficacy, like BMI, breathing or stone migration.
